# Fructose Induces Pulmonary Fibrotic Phenotype Through Promoting Epithelial-Mesenchymal Transition Mediated by ROS-Activated Latent TGF-β1

**DOI:** 10.3389/fnut.2022.850689

**Published:** 2022-05-27

**Authors:** Xiaoxiao Xu, Chuang Ma, Hang Wu, Yuanqiao Ma, Zejin Liu, Peijie Zhong, Chaolei Jin, Wenjuan Ning, Xiao Wu, Yijie Zhang, Jichang Han, Junpeng Wang

**Affiliations:** Infection and Immunity Institute and Translational Medical Center of Huaihe Hospital, Henan University, Kaifeng, China

**Keywords:** fructose, pulmonary fibrosis, epithelial-mesenchymal transition, latent TGF-β, ROS

## Abstract

Fructose is a commonly used food additive and has many adverse effects on human health, but it is unclear whether fructose impacts pulmonary fibrosis. TGF-β1, a potent fibrotic inducer, is produced as latent complexes by various cells, including alveolar epithelial cells, macrophages, and fibroblasts, and must be activated by many factors such as reactive oxygen species (ROS). This study explored the impact of fructose on pulmonary fibrotic phenotype and epithelial-mesenchymal transition (EMT) using lung epithelial cells (A549 or BEAS-2B) and the underlying mechanisms. Fructose promoted the cell viability of lung epithelial cells, while N-Acetyl-l-cysteine (NAC) inhibited such. Co-treatment of fructose and latent TGF-β1 could induce the fibrosis phenotype and the epithelial-mesenchymal transition (EMT)-related protein expression, increasing lung epithelial cell migration and invasion. Mechanism analysis shows that fructose dose-dependently promoted the production of total and mitochondrial ROS in A549 cells, while NAC eliminated this promotion. Notably, post-administration with NAC or SB431542 (a potent TGF-β type I receptor inhibitor) inhibited fibrosis phenotype and EMT process of lung epithelial cells co-treated with fructose and latent TGF-β1. Finally, the fibrosis phenotype and EMT-related protein expression of lung epithelial cells were mediated by the ROS-activated latent TGF-β1/Smad3 signal. This study revealed that high fructose promoted the fibrotic phenotype of human lung epithelial cells by up-regulating oxidative stress, which enabled the latent form of TGF-β1 into activated TGF-β1, which provides help and reference for the diet adjustment of healthy people and patients with fibrosis.

## Introduction

Idiopathic pulmonary fibrosis (IPF) is a chronic, progressive idiopathic lung disease that destroys the dynamic balance of alveolar epithelial cells by endogenous or microenvironmental stimulation, resulting in abnormal repair of alveolar epithelial cells (1, 2). However, there is no specific treatment for IPF, and the two approved drugs (nintedanib and pirfenidone) have limited therapeutic effects. Thus, daily dietary intervention is critical in preventing chronic diseases. Pulmonary fibrosis has been characterized by progressive deposition of extracellular matrix (ECM) proteins such as collagen and fibronectin (3, 4). Epithelial-mesenchymal transition (EMT) has been identified in the alveolar and airway epithelium. One of the driving forces behind various series of fibrosis events is EMT, which is a process in which epithelial cells gain interstitial morphology by increasing the expression of interstitial markers and decreasing the expression of epithelial markers (4, 5). The EMT process is accompanied by down-regulated epithelial markers such as E-cadherin and up-regulated mesenchymal markers including vimentin, N-cadherin, α-smooth muscle actin (α-SMA), and collagen (6, 7). EMT can be induced by various extracellular media alone or in combination, such as TGF-β, epidermal growth factor, fibroblast growth factor-2, insulin-like growth factor-2, Wnt ligand, and hepatocyte growth factor (8, 9).

TGF-β, a multifunctional cytokine, regulates the morphogenesis and differentiation of tissues by affecting cell proliferation, differentiation, apoptosis, and ECM production (10). TGF-β is considered the “master switch” to induce fibrosis in many tissues, such as the lung, and it is involved in many core processes of pulmonary fibrosis, such as epithelial injury, proliferation, and differentiation of myofibroblasts and production of ECM (6, 11). Unlike most growth factors, TGF-β1 is deposited in the ECM as a latent complex (12). It is known that αv integrin, low pH, thrombospondin-1, and reactive oxygen species (ROS) can activate the latent form of TGF-β1. ROS can activate latent TGF-β1 (LTGF-β1) through direct oxidation of latency-related peptide (LAP) or indirect activation of matrix metalloproteinase-2 (MMP-2) and MMP-9 (12, 13).

Additionally, TGF-β1 is the primary inducer of EMT in development, carcinogenesis, and fibrosis (6). It can change the morphology of human lung epithelial cells from oval (epithelial morphology) to fusiform (interstitial morphology) (14). TGF-β1-induced lung epithelial cells, such as A549 or BEAS-2B cells, are usually used as a model for constructing the lung fibrotic phenotype and EMT.

With the significant changes in the human diet, high sugar, high salt, and high-fat intakes increase the incidence of chronic inflammatory diseases (15). For example, high salt intake increases autoimmune diseases, while high glucose intake promotes mitochondria to produce oxidized free radicals, which worsens autoimmune diseases such as inflammatory bowel diseases and experimental autoimmune encephalomyelitis (EAE) (16–18). The main carbohydrate in the typical western diet is fructose, consumed in sucrose (50% fructose) or high fructose corn syrup (42% or 55% fructose). Currently, teenagers consume more than 72.8 grams (12.1% of total calories) of fructose per day (19). 25% or more of the total calories consumed by 20% of teenagers are fructose (20). Americans consume 180 grams of sugar a day, half of which is fructose (21). Excessive fructose intake can lead to metabolic syndrome, including insulin resistance, impaired glucose tolerance, hyperinsulinemia, hypertriglyceridemia, and hypertension (22–25). Fructose also can induce oxidative stress, inflammation, and fibrosis in the liver, heart, and kidney and the growth and metastasis of pancreatic tumors (26–30). However, it is still unclear whether fructose contributes to the development of lung-related diseases such as IPF.

Thus, in this study, we used the TGF-β1-induced lung epithelial cell model *in vitro* to explore the effect of fructose on the lung fibrosis and EMT process and the underlying mechanism.

## Materials and Methods

### Materials

D-Fructose (Fru, #F108335) and N-Acetyl-l-cysteine (NAC, #A105422) were from Shanghai Aladdin. SB431542 (HY-10431) was obtained from MCE. Recombinant human latent TGF-β1 (LTGF-β1, #299-LT) and human TGF-β1 (#240-B) were purchased from R&D Systems. Nitrocellulose membranes (#66485) were obtained from Pall. DCFH-DA (#MX4802) and MitoSOX™ Red mitochondrial superoxide indicator (#M36008) were purchased from Shanghai Maokang and ThermoFisher, respectively.

### Cell Culture

A549 cells (acquired from ATCC) and BEAS-2B cells (acquired from meilunbio®, Dalian, China) were grown in DMEM (Solarbio®, Beijing, China) medium with 1000 mg/L D-Glucose and 10% fetal bovine serum (Biological Industries, Israel) and cultured in an incubator with a temperature of 37°C and 5% CO_2_. After starvation for 24 h, A549 cBEAS-2B ells were treated at the indicated conditions to evaluate cell viability, fibrotic phenotype, and EMT-related protein with or without TGF-β1 (5 ng/ml) or latent TGF-β1 (10 ng/ml) under different fructose concentrations (0, 10, 25 and 50 mM) with or without N-Acetyl-l-cysteine (NAC, 5 mM) (Aladdin, Shanghai, China), or SB431542 (5 μM) to induce the fibrotic phenotype of A549 cells.

### Cell Viability Assay

A549 and BEAS-2B cells were inoculated in a 96-well round plate at 1 × 10^4^ cells/well density. After cells starved for 24 h, we added fructose at the indicated concentration (0, 10, 25, and 50 mM) with or without NAC (5 mM) into each well for 24 h to evaluate the cell viability. Finally, the CCK-8 solution was added to each well and then incubated at 37°C for another 1 h. We used a microplate reader (SpectraMax i3x, Molecular Devices, US) to obtain the absorbance value at 450 nm. The formula of cell viability is as follows: [(absorbance of drug-treated sample – absorbance of Blank group) / (absorbance of control sample – absorbance of Blank group)] ×100%.

### Latent TGF-β1 or TGF-β1 Measurement

After A549 cells were treated with the indicated fructose with latent TGF-β1 at 10 ng/ml for 24 h, the cell-free supernatants were collected to the latent TGF-β1 or TGF-β1 concentration using the Human latent TGF-β1 ELISA^BASIC^ kit (#3550-1H, Mabtech, Sweden) or the Human/Mouse TGF-β1 ELISA kit (#88-8350, eBioscience, San Diego, CA, USA), respectively, following the manufacturer's instructions.

### Wound-Healing Assay

After A549 cells were inoculated in a 6-well plate to ensure that the cells fused up to more than 90% before scratching, we used the sterile 200 μl straw tip to draw three parallel lines in each petri dish and then treated the cells under the indicated conditions for 24 h. Finally, we utilized the inverted light microscope to observe the scratches and obtain images.

### Transwell Assay

Transwell analysis was performed using a Transwell chamber (Corning). Briefly, we inoculated A549 cells in the upper chamber with different reagents at the indicated conditions into a serum-free DMEM medium and added a DMEM medium with 10% FBS into the lower chamber. The cells on the top side of the chamber were removed after being cultured for 24 h; migrated cells from the bottom surface of the upper chamber were fixed with 4% paraformaldehyde solution for 30 min and subsequently stained with 0.1% crystal violet solution for 30 min. Finally, an inverted microscope was utilized to photograph migrating cells, and then the cell numbers were counted in four random fields of vision, with a magnification of ×100.

### Western Blot Assay

After the cells were treated with different reagents at the indicated concentration for 24 h, they were washed with cold PBS, and then total proteins were extracted by RIPA lysis buffer containing PMSF and phosphatase inhibitors. After that, BCA was used to quantify the protein concentration. The same amount of protein was added to the SDS-PAGE gel for electrophoresis and transferred to nitrocellulose. After the nitrocellulose membrane was blocked with 5% skim milk for 1 h, the following primary antibodies: β-actin (1:10,000, Sigma-Aldrich), α-SMA (1:1,000, Sino Biological Inc., Beijing, China), type I collagen (1:1,000, Bioss, Beijing, China), E-cadherin (1:1,000), N-cadherin (1:1,000), vimentin (1:1,000, Snail (1:1,000), p-Smad3 (1:1,000), and Smad3 (1:1,000) (all from Cell Signaling Technology, Inc., Rosemont, IL) were incubated on a shaker at 4°C overnight. Then, the second antibody (goat anti-rabbit or goat anti-mouse horseradish peroxidase binding antibody) was incubated at room temperature for 1 h. The protein bands were visualized by a chemiluminescence gel imaging system (Universal Hood II, Bio-Rad, Hercules, CA). ImageJ analyzed the protein bands to detect the expression level of the target protein.

### Immunofluorescence Staining

After A549 cells were incubated at the indicated conditions, they were fixed with 4% paraformaldehyde, permeabilized with 0.1% Triton X-100 in PBS, blocked with 0.5% FBS in PBS, and incubated with specific primary antibodies α-SMA (1:500) or vimentin (1:200) overnight at 4°C. Then, they were incubated with FITC-conjugated anti-rabbit IgG (Solarbio, Beijing, China). The nucleus was stained with DAPI (Biolegend, San Diego, CA). The fluorescence intensity was observed, and the image was obtained by fluorescence microscope (Olympus, Tokyo, Japan).

### Hydroxyproline (HYP) Evaluation

A549 cells were treated under the indicated conditions, and then the cell-free supernatant was collected, and the HYP content was determined by a hydroxyproline kit (Nanjing Jiancheng Bioengineering Institute, Nanjing, China).

### ROS Measurement

A549 cells were seeded (1 × 10^4^ cells/well) in a 96-well plate and incubated overnight to measure total and mitochondrial ROS in the cells. After the cells were incubated with the indicated concentrations of fructose (0, 10, 25, and 50 mM) for 24 h, they were treated with DCFH-DA for 20 min or MitoSOXRed for 10 min at 37°C in the dark. Cell fluorescence was taken by fluorescence microscope (Olympus, Tokyo, Japan), and a microplate reader read the optical density (OD) value. The formula of DCFH-DA and MitoSOX value is as follows: (OD value of fructose-treated group)/(OD value of control group).

### Statistical Analysis

Mean ± SEM expresses data. We utilized one-way analysis of variance (ANOVA) to analyze the statistical data utilizing prism 9.0 software (Graphpad software) followed by Tukey's test. The difference was set to be statistically significant at *P* < 0.05.

## Results

### Fructose Promotes the Cell Viability of lung epithelial Cells

**Figure 1:**
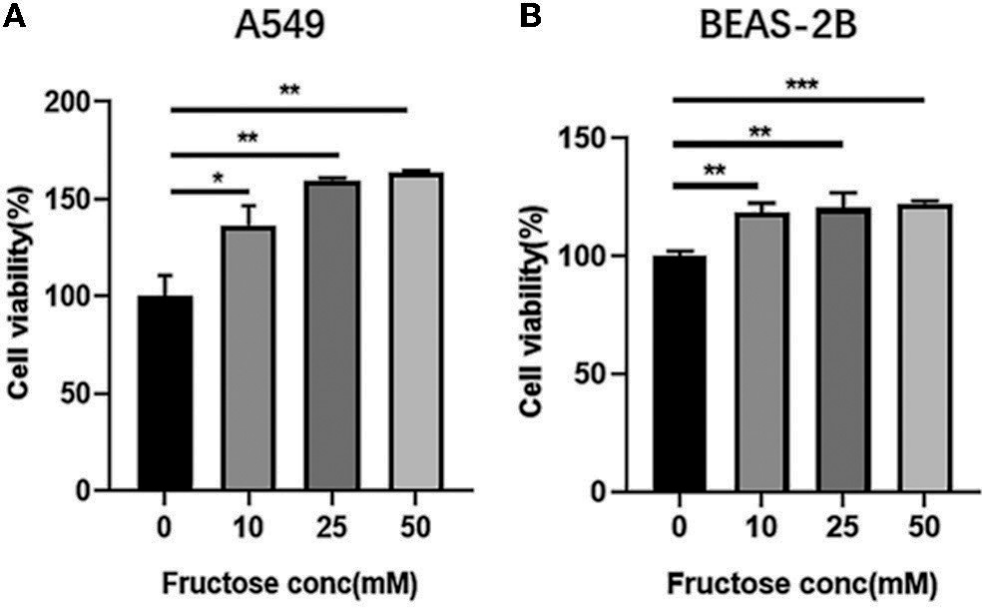
Fructose promotes the cell viability of lung epithelial cells. A549 **(A)** and BEAS-2B **(B)** cells were cultured with different concentrations of fructose (0, 10, 25, and 50 mM) for 24 h, and then a CCK-8 assay was performed to detect the cell viability. Data were expressed as Mean ±S.E.M (*n* = 3) and analyzed using one-way ANOVA followed by Tukey's test. *p < 0.05, **p < 0.01, and ***p < 0.001 vs. 0 mM fructose.

To explore the effect of fructose on the proliferation of A549 and BEAS-2B cells, we used different concentrations of fructose (0, 10, 25, and 50 mM) to treat A549 cells and then evaluated cell viability using a CCK-8 colorimetric assay. As shown in Figure 1A, fructose at 10, 25, and 50 mM increased the viability of A549 cells compared with 0 mM fructose, but no dose-dependent effect was observed among three doses (10, 25, and 50 mM) in A549 cells. Meanwhile, fructose also enhanced human bronchial epithelial BEAS-2B cell viability (Figure 1B).

### Fructose Impacts the Morphological Changes of A549 Cells Induced by Latent TGF-β1

**Figure 2:**
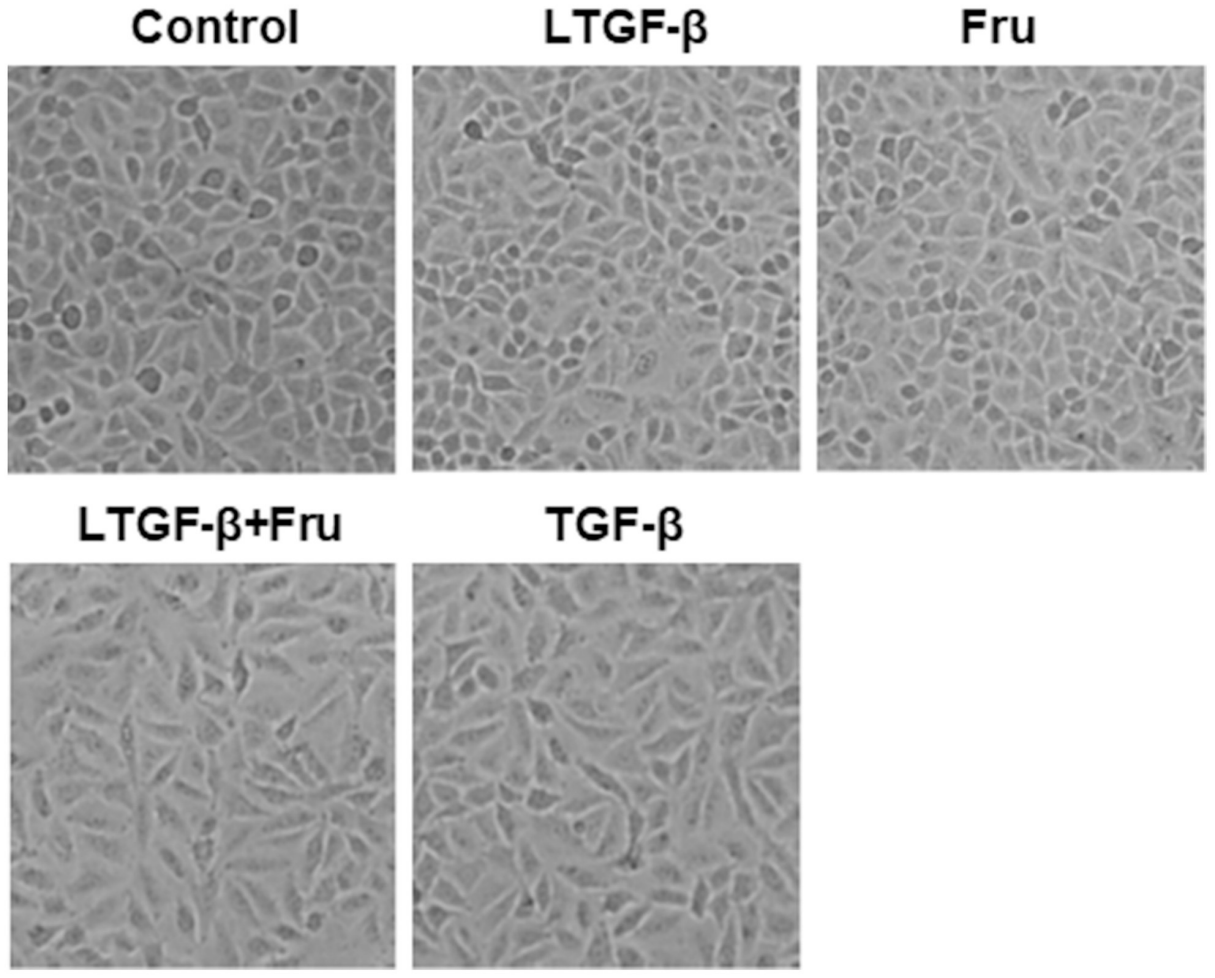
The effect of fructose on the morphology of A549 cells treated with latent TGF-β1. Images are representative pictures of the A549 cells treated with the indicated condition. These experiments were repeated three times. Control, 0 mM fructose; Fru, 10 mM fructose; LTGF-β1, latent TGF-β1; LTGF-β1+Fru, latent TGF-β1 + 10 mM fructose.

TGF-β1, the most potent fibrogenic mediator to induce pulmonary fibrosis, is produced on the alveolar surface extracellular matrix in a latent form (latent TGF-β1) (31). To explore the effect of fructose on the morphology of A549 cells induced by latent TGF-β1, we cultured A549 cells at the indicated condition for 24 h and then observed the morphological changes of A549 cells under an optical microscope. Compared to the control group, TGF-β1 significantly altered the cell morphology (fusiform and long spindle fibrosis phenotype) (Figure 2), but fructose administration or latent TGF-β1 did not affect cell morphology. Although latent TGF-β1 did not affect A549 cell morphology, it induced the cell morphological changes post-supplementation with fructose at 10 mM, similar to TGF-β1-treated A549 cells. The results showed that the co-culture of latent TGF-β1 and fructose could cause the morphology of A549 cells to be fibrotic.

### Fructose Increases the Expression of Fibrosis-Related Proteins in Lung Epithelial Cells-Induced by Latent TGF-β1

**Figure 3:**
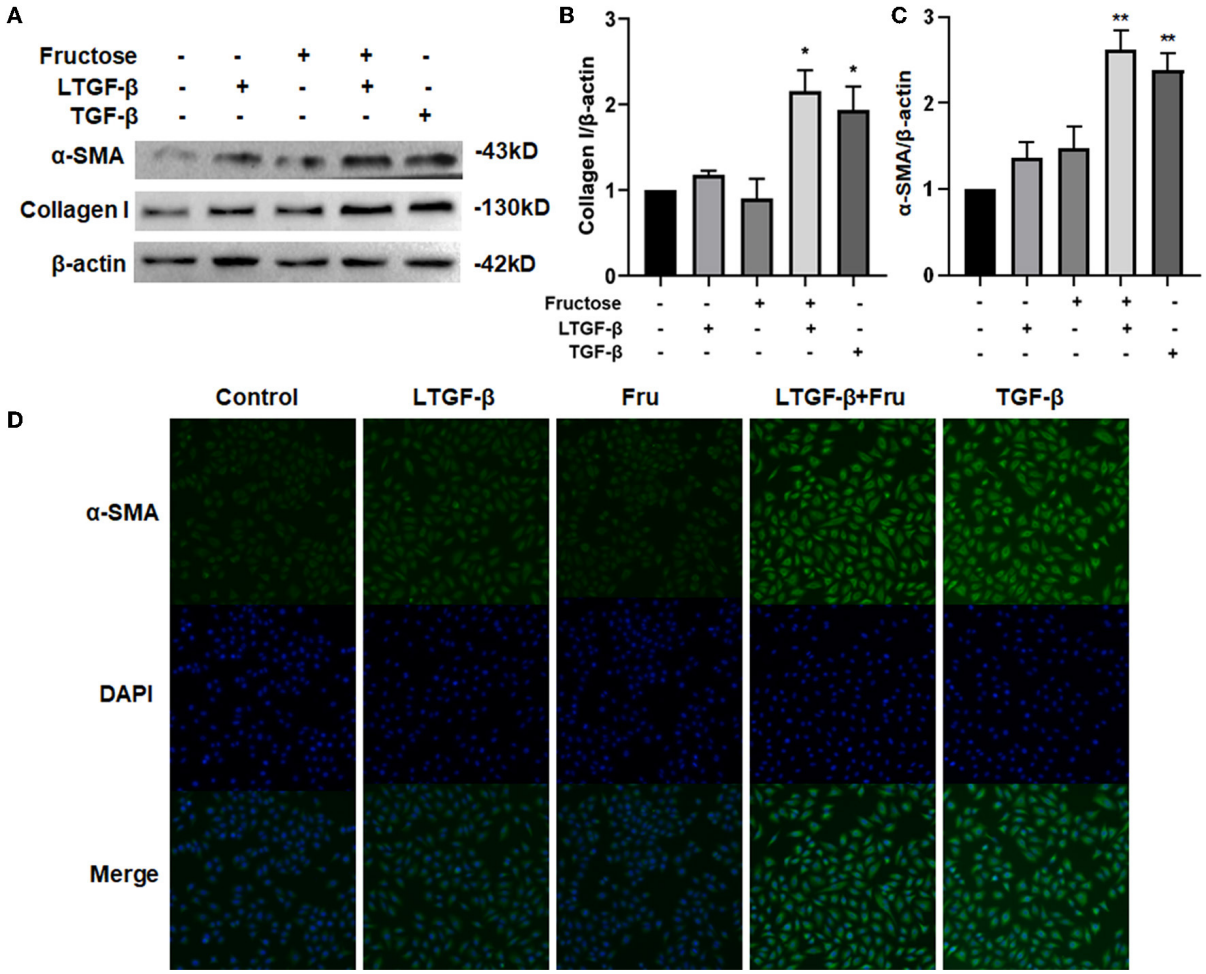
Effect of fructose on fibrosis-related proteins in A549 cells induced by latent TGF-β1. **(A–C)** Expression of collagen I **(A,B)** and α-SMA **(A,C)** in the lysates of A549 cells treated under the indicated condition was analyzed by Western blot. Representative gels were shown, and data were summarized from three independent experiments and analyzed using one-way ANOVA followed by Tukey's test. The relative value was set as fold induction to the control set at 1, normalized to β-actin. **(D)** Additionally, the α-SMA expression was determined by immunofluorescence (200×). * *p* < 0.05, ** *p* < 0.01 vs. control group. Control, 0 mM fructose; Fru, 10 mM fructose; LTGF-β1, latent TGF-β1; LTGF-β1+Fru, latent TGF-β1 + 10 mM fructose.

To further explore whether co-administration with fructose and latent TGF-β1 can cause the fibrotic phenotype of A549 cells, we detected the expressions of fibrosis-related proteins, including collagen I and α-SMA, by Western blot. TGF-β1 significantly increased the expression of collagen I and α-SMA compared with the control group, but latent TGF-β1 or fructose alone did not change these protein expressions (Figures 3A–C). Interestingly, with fructose, latent TGF-β1 up-regulated fibrosis-related proteins such as collagen I and α-SMA. Similar findings were also found in BEAS-2B cells (Supplementary Figure S1). Finally, we used immunofluorescence staining for α-SMA to confirm these results in A549 cells (Figure 3D). However, our preliminary study did not find any difference in fibrosis-related proteins between TGF-β1 and co-treatment with fructose and TGF-β1 (data not shown).

### Fructose Enhances the EMT Process of Lung Epithelial Cells Induced by Latent TGF-β1

**Figure 4:**
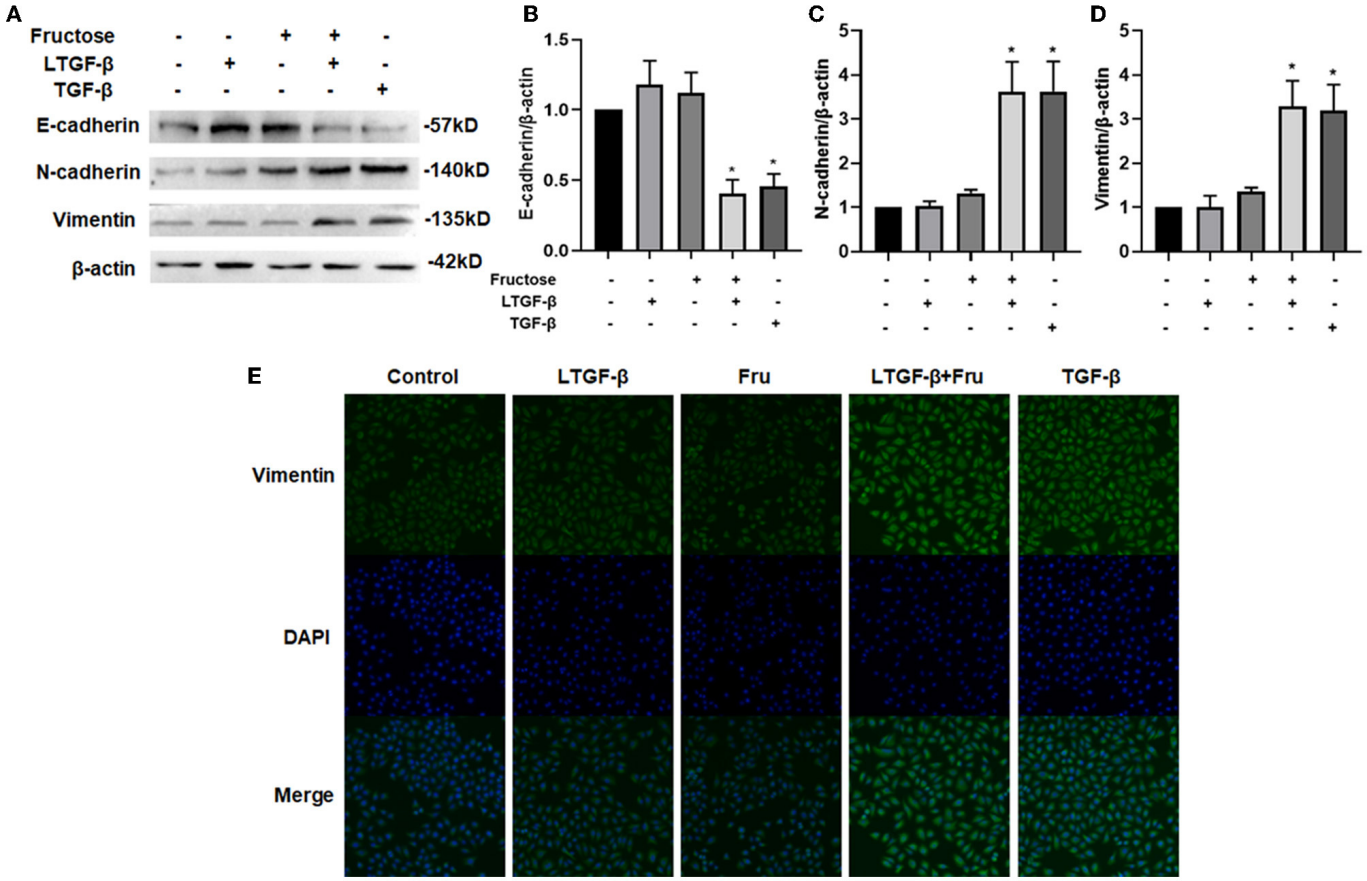
Effect of fructose on the latent TGF-β1-induced EMT process in A549 cells. Expression of E-cadherin, **(A and B)** N-cadherin, **(A and C)** and Vimentin **(A and D)** in the lysates of A549 cells treated under the indicated condition was assessed by Western blot. Representative gels were shown, and data were presented as the Mean ±S.E.M (*n* = 3) and analyzed using one-way ANOVA followed by Tukey's test. The relative value was set as fold induction to the control set at 1, normalized to β-actin. **(E)** Additionally, the vimentin expression was determined by immunofluorescence (200×). * *p* < 0.05, vs. control group. Control, 0 mM fructose; Fru, 10 mM fructose; LTGF-β1, latent TGF-β1; LTGF-β1 + Fru, latent TGF-β1 +10 mM fructose.

The EMT is epithelial cells transforming into stromal cell phenotypes (32). To study the effect of fructose on the latent TGF-β1-induced EMT process of A549 cells, we measured EMT-related protein expression by Western blot. Data showed that co-administration of latent TGF-β1 and fructose resulted in a decreased expression of the epithelial marker E-cadherin while an increase of the mesenchymal markers N-cadherin and vimentin (Figures 4A–D), which was observed in TGF-β1-induced A549 cells. However, compared to the control group, we did not find any difference in these protein expressions from fructose or latent TGF-β1 alone treatment. Finally, we used immunofluorescence to confirm this effect on the vimentin change (Figure 4E). This result was further confirmed in BEAS-2B cells (Supplementary Figure S2).

### Fructose Increases the Level of ROS in A549 Cells in a Dose-Dependent Manner

**Figure 5:**
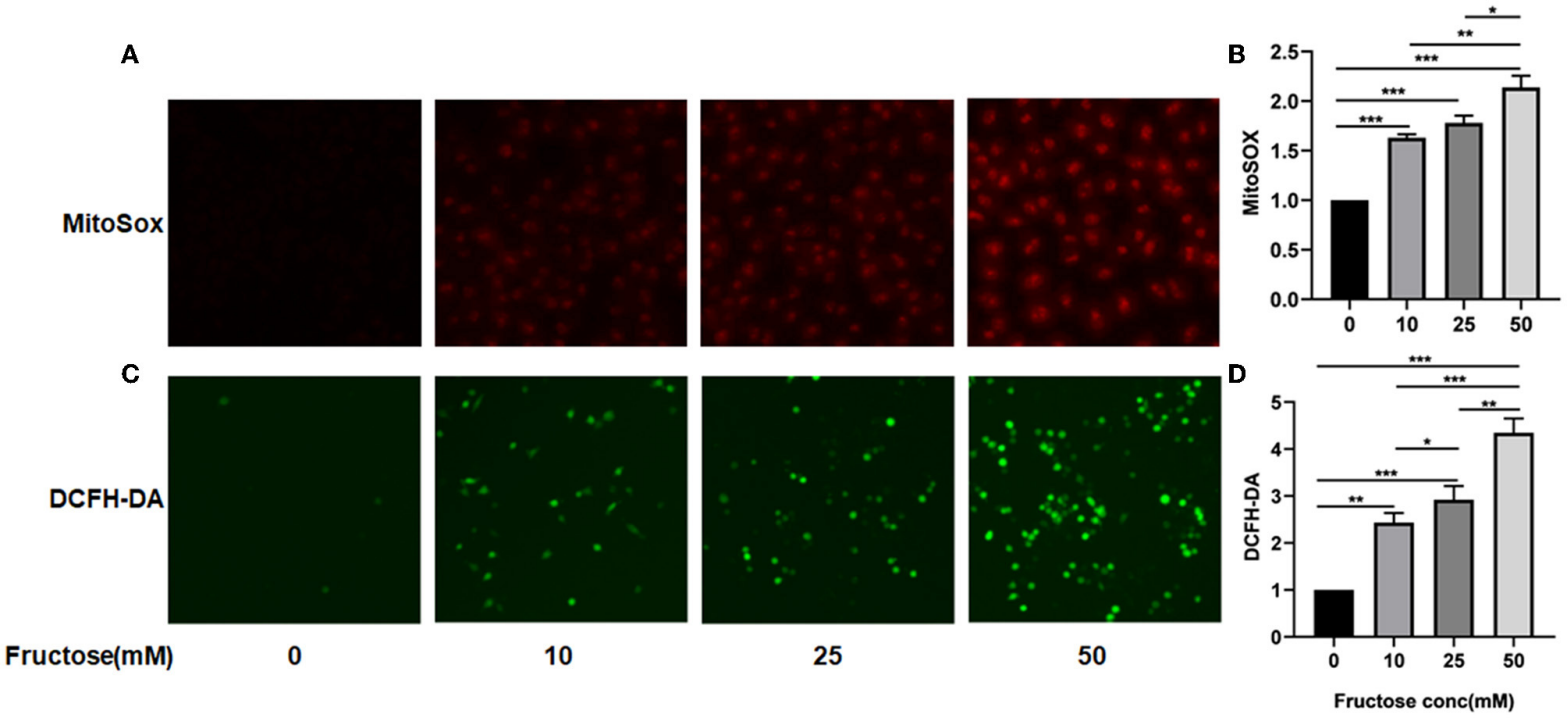
Effects of fructose on total and mitochondria ROS levels in A549 cells. A549 cells were cultured with different concentrations of fructose (0, 10, 25, and 50μM) for 24 h and then were stained with MitoSOX and DCFH-DA, respectively, as described in the “Materials and Methods” section. The morphology of cells was observed under a fluorescence microscope, and representative images for total and mitochondria ROS are represented in **(A,C)**, respectively. Additionally, the absorbance value of MitoSOX **(B)** and DCFH-DA **(D)** was obtained using the enzyme meter. The value of MitoSOX and DCFH-DA **(D)** is obtained by the following formula: (OD value of fructose-treated group)/ (OD value of control group). One-way ANOVA followed by Tukey's test was used for comparison between groups. These experiments were repeated three times. *p < 0.05, ** *p* < 0.01, and *** *p* < 0.001.

Protein fucosylation of fructose in cells can produce ROS (33, 34), which can activate TGF-β1 by oxidation of the latent TGF-β1 form *in vitro* (13). Since we have found that the co-culture of fructose and latent TGF-β1 could promote the fibrosis phenotype of A549 cells, we hypothesized that fructose might increase the production of ROS in cells and then activate TGF-β1. We cultured A549 cells with different fructose concentrations (0, 10, 25, and 50 mM) to verify the above hypothesis. As shown in Figure 5, fructose increased total and mitochondrial ROS levels in A549 cells in a dose-dependent manner.

### NAC Inhibits the Fibrotic and EMT Process Lung Epithelial Cells Induced by Fructose and Latent TGF-β1

**Figure 6:**
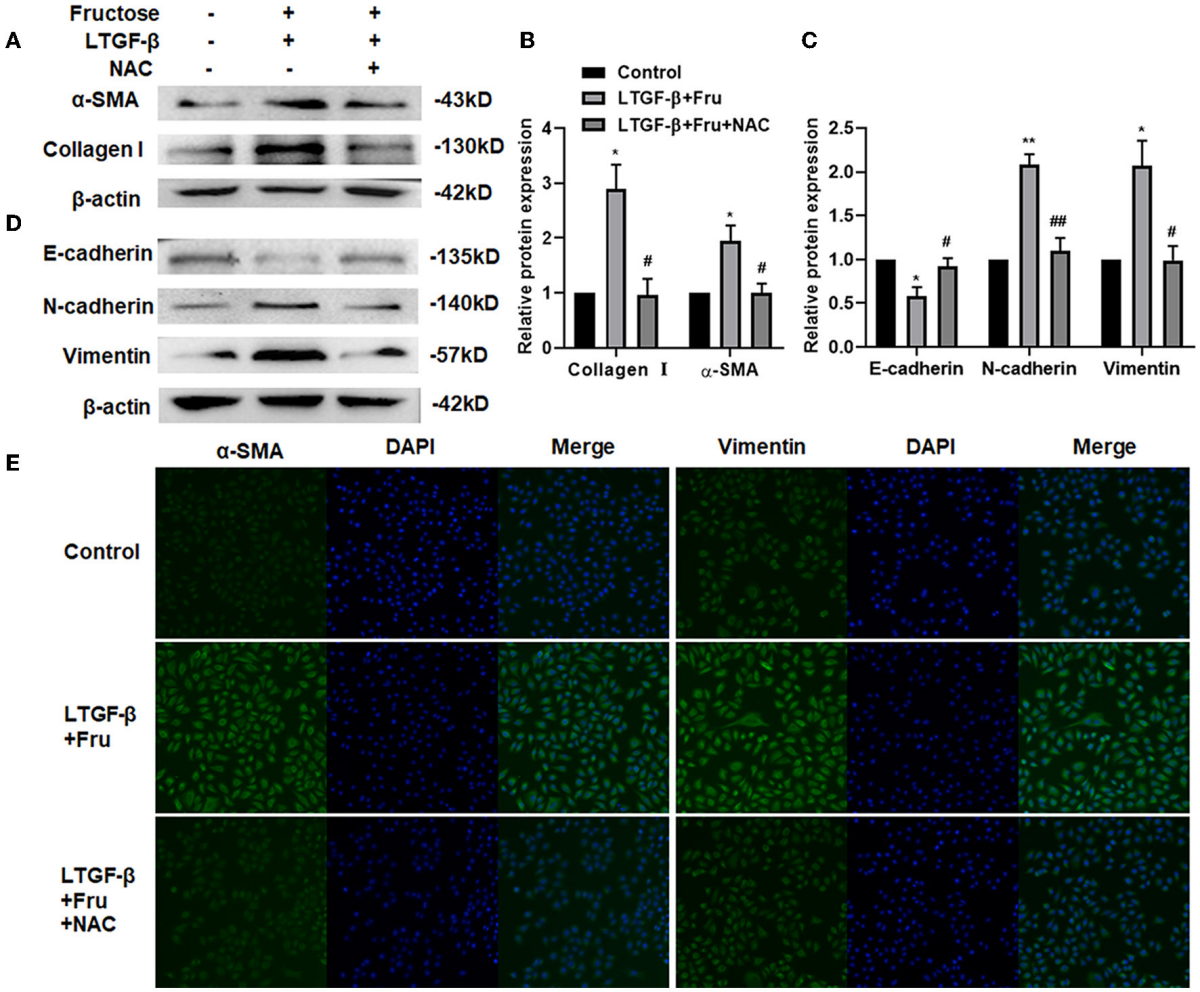
Effect of NAC scavenging fructose-induced ROS on the fibrosis and EMT process of A549 cells treated by latent TGF-β1. A549 cells were treated under the indicated treatment for 24 h, and then the expression of fibrosis-related proteins **(A and B)** (Collagen 1 and α-SMA) and EMT-related proteins **(C and D)** (N-cadherin, E-cadherin, and Vimentin) were assessed by Western blot. Data are expressed as the Mean ± S.E.M (*n* = 3) and analyzed using one-way ANOVA followed by Tukey's test. The relative value was set as fold induction to that of the control set at 1, normalized to β-actin. *p < 0.05 and ** *p* < 0.01 vs. control group; ^#^*p* < 0.05, ^##^*p* < 0.01, compared with the LTGF-β1 + Fru group. Additionally, the vimentin or α-SMA expression was determined by immunofluorescence (200×). **(E)** Control, 0 mM fructose; Fru, 10 mM fructose; LTGF-β1+Fru, latent TGF-β1 + 10 mM fructose; NAC, N-Acetyl-l-cysteine.

NAC is a potent antioxidant with a free radical scavenging function, removing ROS, ROS's precursors, and inhibiting ROS generation (35, 36). To further verify whether ROS produced by fructose is involved in the activation of TGF-β1, we added NAC to the cells treated with fructose and latent TGF-β1 and then detected the expression of fibrosis- and EMT- related proteins. Compared with the latent TGF-β1 + fructose group, administration of NAC suppressed the unregulated expression of fibrosis-related proteins collagen I and α-SMA (Figures 6A,B). Meanwhile, mesenchymal markers N-cadherin and vimentin decreased significantly, while the expression of epithelial markers E-cadherin increased in the presence of NAC (Figures 6C,D). This result was also confirmed in BEAS-2B cells (Supplementary Figure S3). The α-SMA and vimentin expression was further verified by immunofluorescence (Figure 6E). The results suggested that NAC could prevent the fibrosis and EMT process of lung epithelial cells induced by fructose and latent TGF-β1 by eliminating ROS.

### TGF-β1 Specific I Receptor Inhibitor Impairs the Effect of Fructose on Fibrotic and EMT Process of A549 Cells Induced by Latent TGF-β1

**Figure 7:**
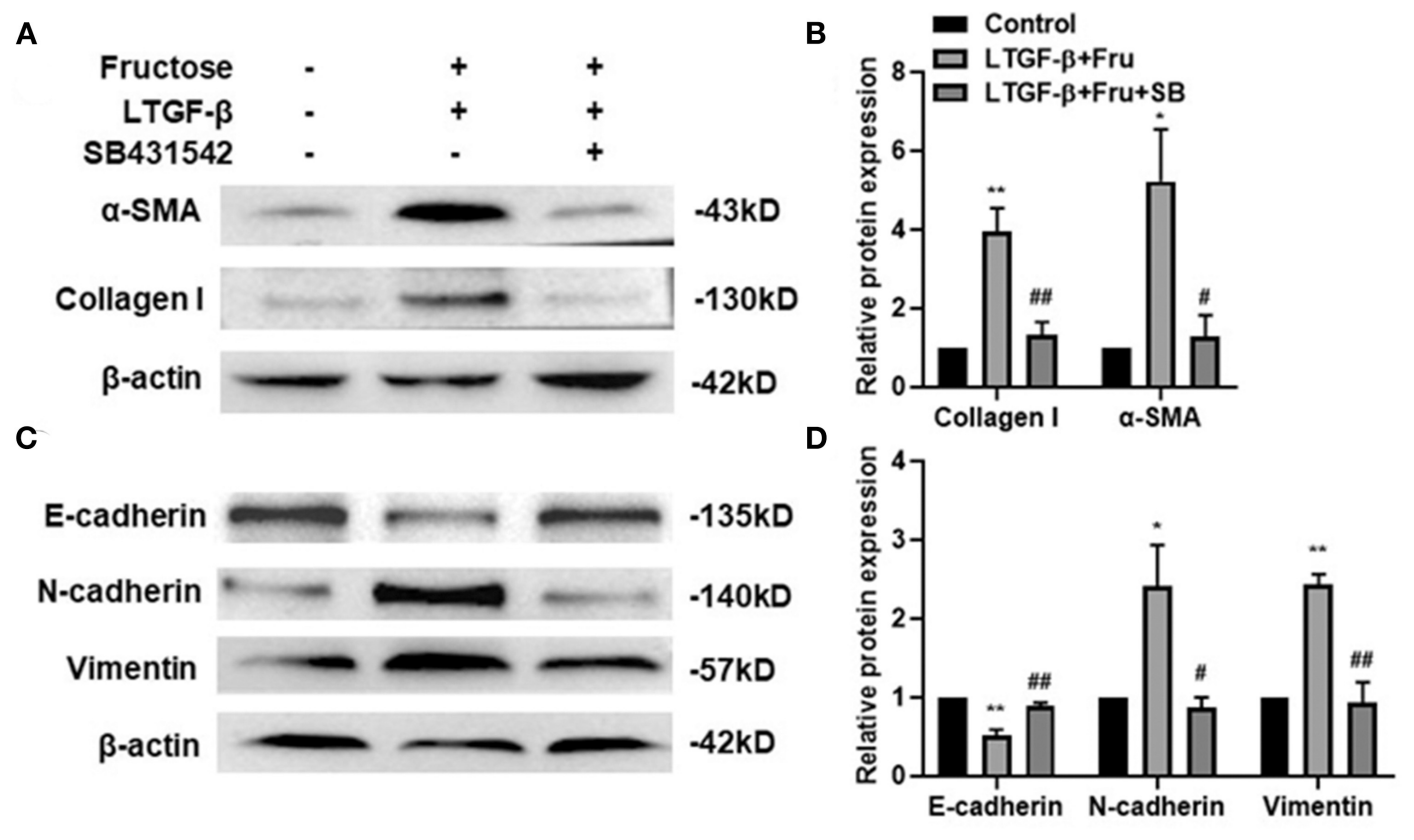
Effect of SB431542 on fibrotic- and EMT- related protein expression of A549 cells induced by fructose and latent TGF-β1. A549 cells were treated under the indicated conditions, and then the expression of collagen I, **(A and B)** α-SMA, **(A and B)** N-cadherin, **(C and D)** E-cadherin, **(C and D)** and vimentin **(C and D)** was assessed by Western blot. Data are set as the Mean ± SEM (*n* = 3). The relative value was set as fold induction to that of the control set at 1, normalized to β-actin. Comparisons between groups were achieved using a one-way ANOVA by Tukey's test. *p < 0.05 and **p < 0.01 vs. control group; ^#^*p* < 0.05, ^*##*^*p* < 0.01, compared with the LTGF-β1+Fru group. Control, 0 mM fructose; LTGF-β1+ Fru, latent TGF-β1 + 10 mM fructose; LTGF-β1+10 mM Fru+SB, latent TGF-β1 + 10 mM fructose + SB431524.

To further confirm whether fructose-generated ROS activates latent TGF-β1 and consequently mediates at least a component of the consequent fibrogenesis and EMT of A549 cells, we added a potent TGF-β type I receptor inhibitor, SB431542, into the A549 cells treated under the indicated conditions and then determined the expression of fibrosis- and EMT-related proteins by Western blot. As expected, administration of SB43152 significantly inhibited the fibrosis-related proteins collagen I and α-SMA and EMT-related proteins, including mesenchymal markers N-cadherin and vimentin, while preventing the downregulation of epithelial markers E-cadherin in the A549 cells co-supplemented with fructose and latent TGF-β1 (Figure 7).

### Effect of Fructose on the Content of Hydroxyproline

**Figure 8:**
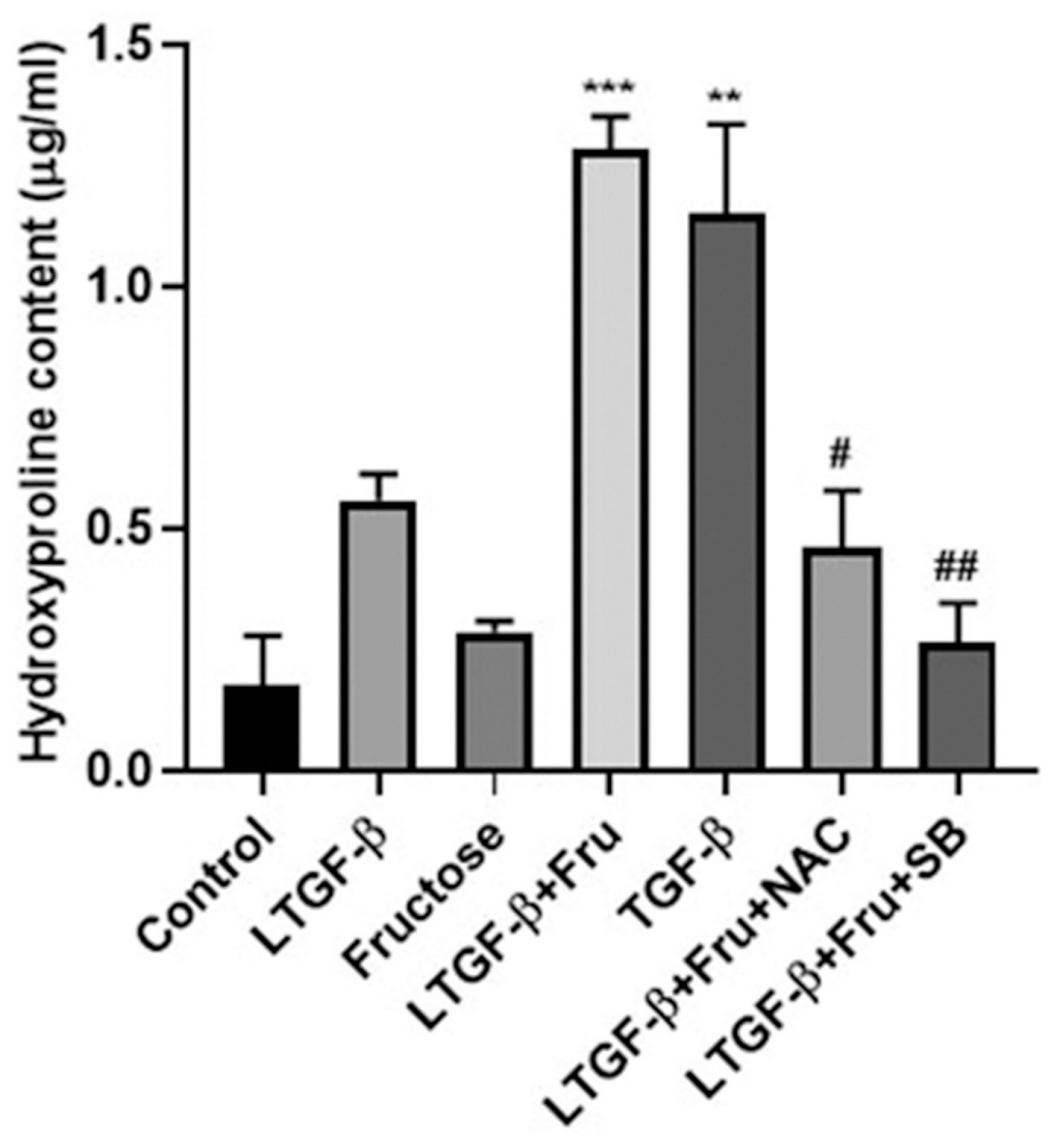
Effect of fructose on the content of hydroxyproline. A549 cells were treated under the indicated conditions, and then the cell supernatant was collected, and the hydroxyproline kit determined the hydroxyproline content. Data are set as the Mean ± SEM (*n* = 3). Comparisons between groups were achieved using a one-way ANOVA followed by Tukey's test. **p < 0.001, ***p < 0.001 vs. control group; ^#^*p* < 0.05, ^*##*^*p* < 0.01, compared with the LTGF-β1+Fru group. Control, 0 mM fructose; LTGF-β1 + Fru, latent TGF-β1 + 10 mM fructose; LTGF-β1+Fru+NAC, latent TGF-β1 + 10 mM fructose + N-Acetyl-l-cysteine; LTGF-β1+Fru+SB, latent TGF-β1 + 10 mM fructose + SB431524.

HYP is one of the amino acids and one of the main components of collagen tissue. We used the HYP kit to verify the above conclusion further to determine the cell supernatant. As shown in Figure 8, the content of HYP in the latent TGF-β1 plus fructose group and TGF-β1 group increased significantly compared with the control group, and the addition of NAC and SB43154 inhibited the production of HYP. The results further confirmed that fructose could induce latent TGF-β1-induced fibrosis in A549 cells, which could be inhibited by scavenging ROS or blocking the TGF-β signal.

### Fructose Promotes the Expression of the TGF-β1/Smad3 Pathway, Snail, and Latent TGF-β1 Converted to TGF-β1 in A549 Cells Treated With Latent TGF-β1

**Figure 9:**
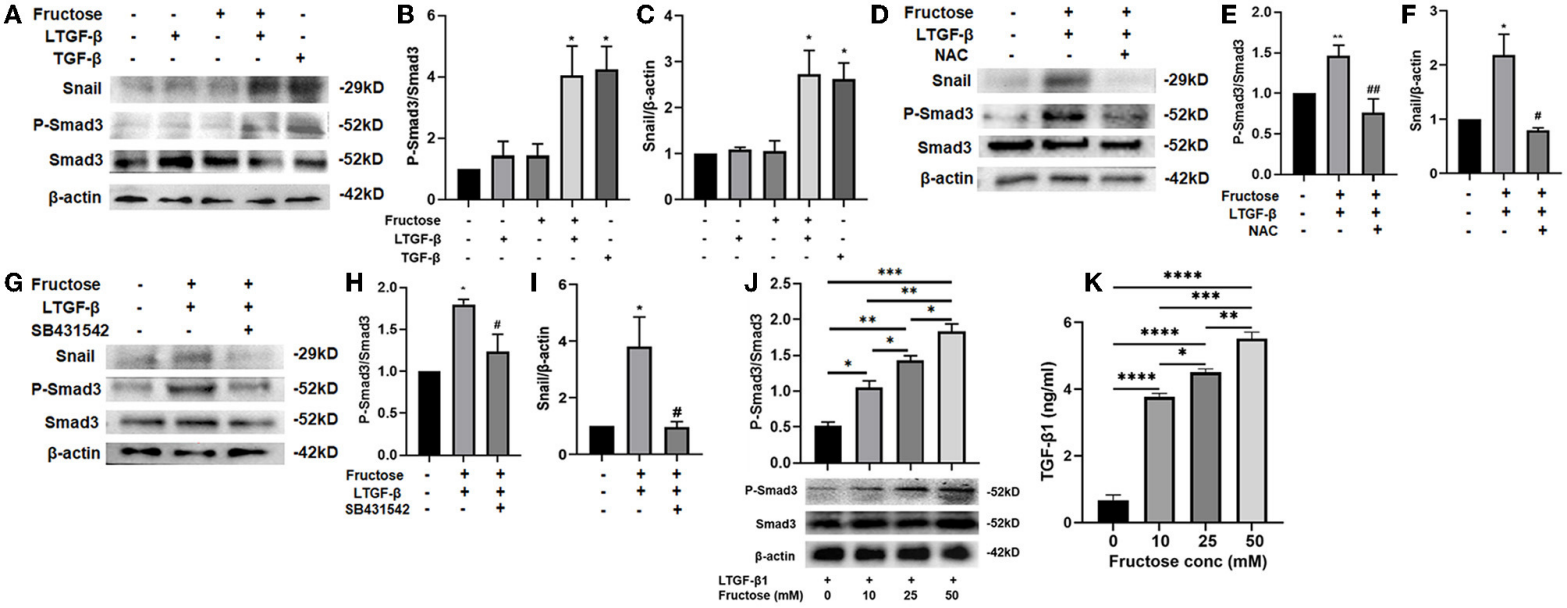
Effect of fructose on the P-Smad3 and Snail expression and latent TGF-β1 converted to TGF-β1 in latent TGF-β1-induced A549 cells. A549 cells were treated under the indicated condition for 24 h, and then western blot analyzed the P-Smad3 and Snail protein levels **(A–J)**, and ELISA measured the leftover latent TGF-β1 level **(K)**. Data are set as the Mean ± S.E.M (*n* = 3). Comparisons between groups were achieved using a one-way ANOVA followed by Tukey's test. *p < 0.05, ** *p* < 0.01, **p < 0.001 vs. the control group; ^#^*p* < 0.05, ^##^*p* < 0.01, compared with the LTGF-β1+10 mM Fructose group. LTGF-β1, latent TGF-β1; NAC, N-Acetyl-l-cysteine.

The TGF-β1 signal pathway is closely related to forming the fibrotic phenotype of A549 cells. Smad3 plays a crucial role in signal activation, and the Snail1 is a downstream transcription factor of Smad3 (32, 37). To further study the underlying mechanism of fructose causing the fibrotic phenotype of A549 cells, we first tested the expression of Smad3 phosphorylation and Snail after A549 cells were treated under the indicated condition. TGF-β1 increased the p-Smad3 and Snail expression compared to the control group, which was also observed in the fructose and latent TGF-β1 co-treated group (Figures 9A–C). However, the upregulation of p-Smad3 and Snail1 in the fructose and latent TGF-β1 group was reversed by administrating NAC (Figures 9D–F) or TGF-β specific I receptor inhibitor SB431542 (Figures 9G–I). Next, we measured the expression of p-Smad3 from A549 cells co-treated with latent TGF-β1 in the different concentrations of fructose and found that fructose dose-dependently increased the phosphorylated Smad3 expression (Figure 9J). Finally, we determined whether fructose could promote the latent TGF-β1 converted to activated TGF-β1. Since we could not detect the TGF-β1 level in the cell-free supernatants from cells co-treated with latent TGF-β1 in different doses of fructose, we speculate that the activated TGF-β1 should be utilized during the fibrotic process of lung epithelial cells. Thus, we directly evaluated the leftover latent TGF-β1 and found that the latent TGF-β1 was undetectable in the absence of latent TGF-β1 or the presence of fructose alone (Data not shown). However, there was 9.336 ng/ml TGF-β1 in the latent TGF-β1 group alone, whereas co-treatment of latent TGF-β1 and fructose at 10, 25, and 50 mM had 6.236, 5.496, and 4.488 ng/ml TGF-β1, indicating that fructose could promote 3.745, 4.324, 5.652 ng/ml latent TGF-β1 converted to activated TGF-β1, respectively, which could be involved in the TGF-β1-induced fibrotic and EMT process of lung epithelial cells (Figure 9K).

### Effect of Fructose on Migration and Invasion Ability of Latent TGF-β1-Induced A549 Cells

**Figure 10:**
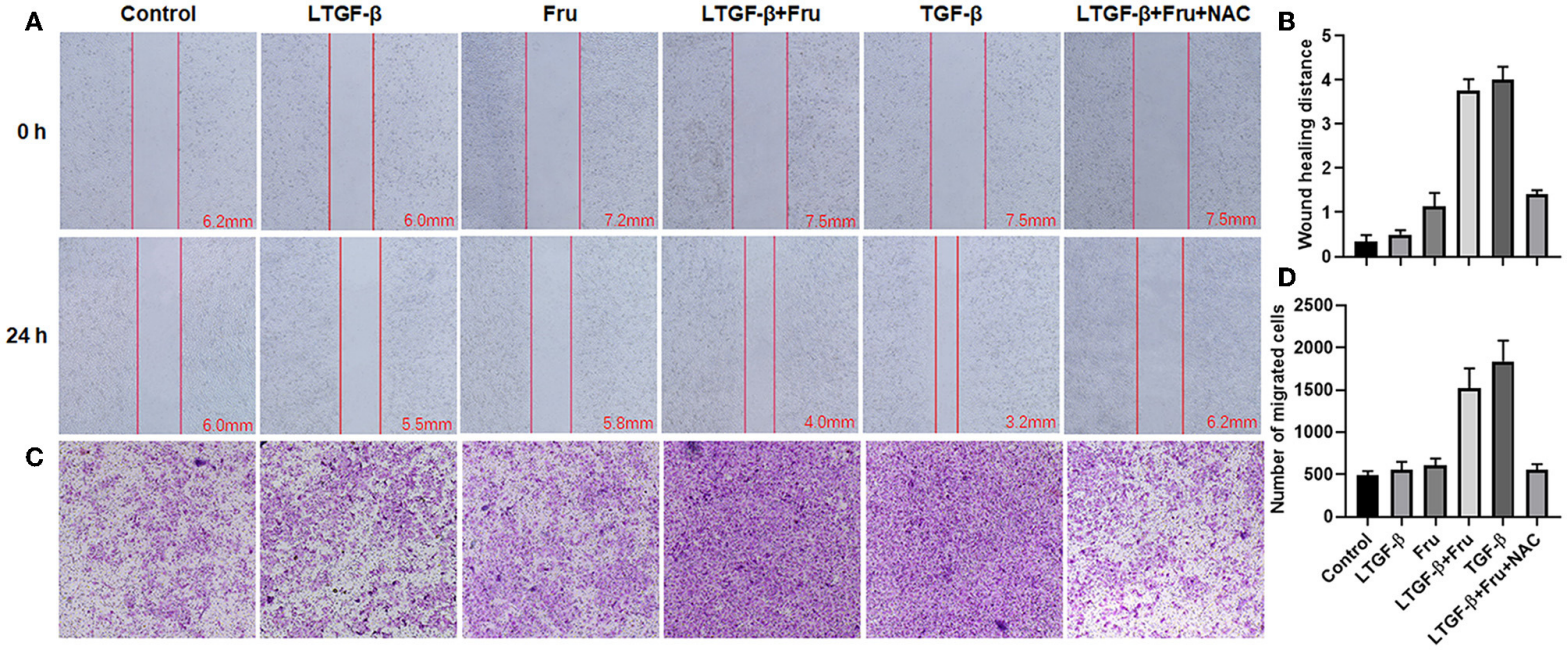
Effect of fructose on latent TGF-β1-induced A549 cell migration and invasion. **(A)** Wound healing assays of A549 cells, the wound closure was photographed at 24 post-scratching (40× magnification). **(B)** Wound healing distance = (the initialized width of the scratch) – (the final width). **(C,D)** Invasion Chambers (40× magnification) and counts of cells under the microscope. Control, 0 mM fructose; Fru, 10 mM fructose; LTGF-β1, latent TGF-β1; LTGF-β1+Fru, latent TGF-β1 + 10 mM fructose; LTGF-β1+Fru+NAC, latent TGF-β1 + 10 mM fructose + N-Acetyl-l-cysteine.

Compared with the control group, cell migration (Figures 10A,B) and invasion (Figures 10C,D) in the TGF-β1 or latent TGF-β1 + fructose group were significantly enhanced, while there was no significant change in cell migration and invasion in the fructose or latent TGF-β1 alone group. However, fructose combined with latent TGF-β1's effect was impaired post-administration with NAC.

## Discussion

IPF is a chronic, progressive idiopathic lung disease that destroys the dynamic balance of alveolar epithelial cells by endogenous or microenvironmental stimulation, resulting in abnormal repair of alveolar epithelial cells (1). EMT actively participates in the progress and maintenance of IPF (25). Diet is considered a potential environmental risk factor for the increased incidence of inflammatory diseases, but its underlying mechanism is unclear (18). Fructose is usually used as a food additive, which has the characteristics of high sweetness. However, studies have found that fructose has many potential hazards to our bodies. e.g., fructose can promote the formation of fatty liver by promoting the synthesis of fat (24, 38) and the growth of the tumor (breast cancer, pancreatic cancer, and small bowel cancer) by increasing the flow of pentose phosphate pathway (30, 39). In this study, we first demonstrated that fructose increased the viability of lung epithelial cells, including A549 and BEAS-2B cells, but could be prevented by administration with NAC. Secondly, fructose at 10 mM significantly resulted in morphological alteration, increased fibrosis- and EMT- related protein expression (α-SMA, collagen I, N-cadherin, and vimentin), invasion and migration, and the decreased epithelial marker E-cadherin expression of lung epithelial cells treated with latent TGF-β1. Mechanism analysis found that fructose-producing ROS mediated latent TGF-β1-induced lung fibrotic phenotype and EMT process, and such effects were prevented post-administration with NAC. Finally, our results reveal that fructose-activated TGF-β1 promoted Smad3 phosphorylation and Snail expression, whereas these effects could be controlled by a TGF-β type I receptor inhibitor.

ROS, a normal byproduct of various cellular processes, can modulate gene expression in growth, development, and apoptosis (40). ROS has a double-edged sword effect on cell metabolism as a signal transducer. e.g., ROS promotes cell growth, invasion, and migration at low to moderate levels (41, 42), while high ROS concentrations result in cell death via direct or indirect mechanisms (43). Fructose has two stereoisomeric forms: linear ketal and fructofuranose (5 membered rings) form. The molecular instability of the five-membered furanose ring can lead to a protein fucosylation reaction and the release of superoxide radical (33, 34). Fructose enters cells through a fructose transporter (GLUT5) and is oxidized and utilized. GLUT5, encoded by SLC2A5, is a unique fructose transporter in mammalian cells. It has been found that the utilization of fructose by cells depends mainly on the expression level of SLC2A5, and fructose has different effects on the viability of different cells (44). Studies have shown that low or moderate ROS can promote cell proliferation (45). In the present study, ROS induced by fructose did not lead to the cell death of human lung epithelial cells (A549 and BEAS-2B) but increased A549 and BEAS-2B cell viability in a non-dose dependent manner since the elimination of ROS by NAC impaired the cell proliferation promoted by ROS-induced by fructose (Supplementary Figure S5). These data suggest that fructose induces moderate ROS levels that promotes human lung cell growth.

TGF-β, a potential inducer of ECM proteins, could be produced by numerous cells in a latent form by binding a latent associated peptide that must be activated by αv integrins, ROS, plasmin, and MMPs for TGF-β1 (46–48) and then initiate its multiple biological functions. Evidence has proved that glucose, another monosaccharide, at 25 mM, can activate latent TGF-β1 for TGF-β1 by an increased ROS in T cells, subsequently promoting the differentiation of Th17 cells in the presence of IL-6 and ultimately resulting in an aggravation of EAE (18). However, glucose could not promote Th17 cell differentiation induced by TGF-β1 and IL-6 (18). Since fructose can produce 100 times more ROS than glucose (21), we speculate that ROS-induced by fructose should activate TGF-β1 by oxidation of the latent associated peptide. As expected, we found that fructose dose-dependently increased total and mitochondria ROS but did not affect cytoplasm ROS production (data not shown), indicating that the production of ROS promoted by fructose might be mainly derived from mitochondria.

Additionally, fructose at 10 mM could also produce more total and mitochondrial ROS than that of the control treatment, which might subsequently initiate TGF-β1 activation. Indeed, when administered with fructose at 10 mM, latent TGF-β1 could induce the morphology change, the expression of fibrosis- and EMT-related proteins of human lung epithelial cells. In contrast, NAC could prevent such alteration, indicating that ROS-induced by fructose treatment at 10 mM can make latent TGF-β1 into activated TGF-β1.

Although it is still unclear for the pathogenesis of pulmonary fibrosis, the TGF-β1 signal pathway is considered the key to the formation of pulmonary fibrosis (37, 49). The activity level of the TGF-β1 signaling pathway is associated with mortality in patients with pulmonary fibrosis. TGF-β1/Smad signal plays a vital role in tumor progression and the EMT process. Studies have shown that the Smad3 signaling pathway is involved in the EMT process of A549 cells induced by TGF-β1 (50). Additionally, the TGF-β1/Snail signal involves the EMT process in pulmonary fibrosis (32). Snail is a zinc finger protein and a regulatory factor of EMT-related gene expression, inhibiting epithelial genes and activating the mesenchymal phenotype, and could be up-regulated in patients with fibrous diseases (14, 51, 52). This study found that co-administration with fructose and latent TGF-β1 up-regulated the Smad3 phosphorylation in a dose-dependent manner and the Snail expression, which the TGF-β type I receptor inhibitor could inhibit. These findings suggested that ROS-activated TGF-β1 involved the TGF-β1-induced fibrotic phenotype and EMT process via TGF-β1/Smad/Snail signals. However, whether dietary fructose influences pulmonary fibrosis *in vivo* needs to be investigated.

**Figure 11:**
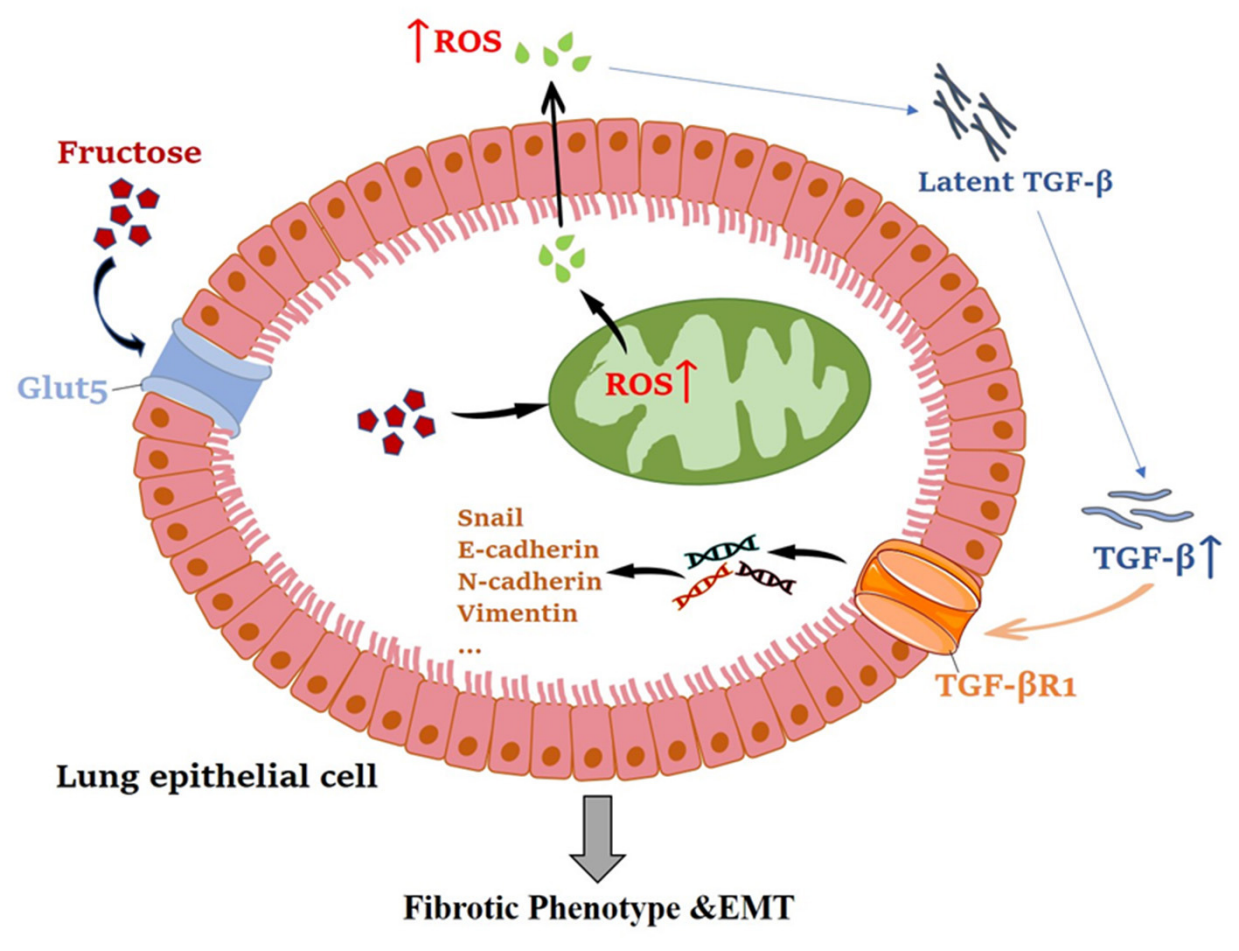
The mechanism of fructose promoting pulmonary epithelial cell fibrosis and EMT.

Overall, this study has shown that fructose can promote latent TGF-β1-induced fibrosis and EMT in human epithelial cells. The mechanisms reveal that fructose's effects may be due to total and mitochondrial ROS accumulation, leading to latent TGF-β1 activation and pulmonary fibrosis (Figure 11). Thus, a deeper understanding of the molecular mechanism of ROS homeostasis defect may provide more options for treating pulmonary fibrosis.

## Data Availability Statement

The original contributions presented in the study are included in the article/Supplementary Material, further inquiries can be directed to the corresponding author/s.

## Author Contributions

JW, XX, JH, and YZ contributed to the design of the study. XX, CM, HW, YM, ZL, PZ, CJ, WN, and XW performed the experiments. XX analyzed the data. XX and JW wrote or critically revised the manuscript. All authors read and approved the final manuscript.

## Funding

This work was supported in part by the National Natural Science Foundation of China (U2004104), the Natural Science Foundation of Henan Province (202300410080), the Science and Technology Development Project of Henan Province (212102310696, 192102310104, 202102310094, and 202102310369), the Key Project of Henan Education Committee (21A310005), the Postgraduate Talent Program of Henan University (SYL20060187 and SYL20060189), and the High-Level Medical Achievement Cultivation Program of Henan University.

## Conflict of Interest

The authors declare that the research was conducted in the absence of any commercial or financial relationships that could be construed as a potential conflict of interest.

## Publisher's Note

All claims expressed in this article are solely those of the authors and do not necessarily represent those of their affiliated organizations, or those of the publisher, the editors and the reviewers. Any product that may be evaluated in this article, or claim that may be made by its manufacturer, is not guaranteed or endorsed by the publisher.

## References

[1] Barratt SL Creamer A Hayton C Chaudhuri N. Idiopathic Pulmonary Fibrosis (IPF): an overview. J Clin Med. (2018) 7:201. 10.3390/jcm7080201 30082599 PMC6111543

[2] Fandiño J Toba L González-Matías LC Diz-Chaves Y Mallo F. GLP-1 receptor agonist ameliorates experimental lung fibrosis. Sci Rep. (2020) 10:18091. 10.1038/s41598-020-74912-1 33093510 PMC7581713

[3] Tjin G White ES Faiz A Sicard D Tschumperlin DJ Mahar A. Lysyl oxidases regulate fibrillar collagen remodelling in idiopathic pulmonary fibrosis. Dis Models Mech. (2017) 10:1301–12. 10.1242/dmm.030114 29125826 PMC5719253

[4] Chen L Li S Li W. LOX/LOXL in pulmonary fibrosis: potential therapeutic targets. J Drug Target. (2019) 27:790–6. 10.1080/1061186X.2018.1550649 30457362

[5] Andugulapati SB Gourishetti K Tirunavalli SK Shaikh TB Sistla R. Biochanin-A ameliorates pulmonary fibrosis by suppressing the TGF-beta mediated EMT, myofibroblasts differentiation and collagen deposition in in vitro and in vivo systems. Phytomedicine. (2020) 78:153298. 10.1016/j.phymed.2020.153298 32781391 PMC7395646

[6] Willis BC Borok Z. TGF-beta-induced EMT: mechanisms and implications for fibrotic lung disease. Am J Physiol Lung Cell Mol Physiol. (2007) 293:L525–534. 10.1152/ajplung.00163.2007 17631612

[7] Kyung SY Kim DY Yoon JY Son ES Kim YJ Park JW. Sulforaphane attenuates pulmonary fibrosis by inhibiting the epithelial-mesenchymal transition. BMC Pharmacol Toxicol. (2018) 19:13. 10.1186/s40360-018-0204-7 29609658 PMC5879815

[8] Xu J Lamouille S Derynck R. TGF-beta-induced epithelial to mesenchymal transition. Cell Res. (2009) 19:156–72. 10.1038/cr.2009.5 19153598 PMC4720263

[9] Das B Sinha D. Diallyl disulphide suppresses the cannonical Wnt signaling pathway and reverses the fibronectin-induced epithelial mesenchymal transition of A549 lung cancer cells. Food and Function. (2019) 10:191–202. 10.1039/C8FO00246K 30516195

[10] Yue J Mulder KM. Transforming growth factor-beta signal transduction in epithelial cells. Pharmacol Ther. (2001) 91:1–34. 10.1016/S0163-7258(01)00143-7 11707292

[11] Sime PJ O'Reilly KM. Fibrosis of the lung and other tissues: new concepts in pathogenesis and treatment. Clin Immunol. (2001) 99:308–19. 10.1006/clim.2001.5008 11358425

[12] Jobling MF Mott JD Finnegan MT Jurukovski V Erickson AC Walian PJ. Isoform-specific activation of latent transforming growth factor β (LTGF-β) by reactive oxygen species. Radiat Res. (2006) 166:839–48. 10.1667/RR0695.1 17149983

[13] Chang CH Pauklin S. ROS and TGFbeta: from pancreatic tumour growth to metastasis. J Exp Clin Cancer Res. (2021) 40:152. 10.1186/s13046-021-01960-4 33941245 PMC8091747

[14] Jiang F Yang Y Xue L Li B Zhang Z. (2017). 1α,25-dihydroxyvitamin D3 Attenuates TGF-and#946;-induced pro-fibrotic effects in human lung epithelial cells through inhibition of epithelial-mesenchymal transition. Nutrients 9:980. 10.3390/nu9090980 28878195 PMC5622740

[15] Lerner A Matthias T. Changes in intestinal tight junction permeability associated with industrial food additives explain the rising incidence of autoimmune disease. Autoimmun Rev. (2015) 14:479–89. 10.1016/j.autrev.2015.01.009 25676324

[16] Kleinewietfeld M Manzel A Titze J Kvakan H Yosef N Linker RA. Sodium chloride drives autoimmune disease by the induction of pathogenic TH17 cells. Nature. (2013) 496:518–22. 10.1038/nature11868 23467095 PMC3746493

[17] Manzel A Muller DN Hafler DA Erdman SE Linker RA Kleinewietfeld M. Role of “Western diet” in inflammatory autoimmune diseases. Curr Allergy Asthma Rep. (2014) 14:404. 10.1007/s11882-013-0404-6 24338487 PMC4034518

[18] Zhang D Jin W Wu R Li J Park SA Tu E. (2019). High glucose intake exacerbates autoimmunity through reactive-oxygen-species-mediated TGF-beta cytokine activation. Immunity. 51:671–81 e675. 10.1016/j.immuni.2019.08.001 31451397 PMC9811990

[19] Vos MB Kimmons JE Gillespie C Welsh J Blanck HM. Dietary fructose consumption among US children and adults: the third national health and nutrition examination survey. Medscape J Med. (2008) 10:160. 18769702 PMC2525476

[20] Bray George A. How bad is fructose? Am J Clin Nutr. (2007) 86:895–6. 10.1093/ajcn/86.4.895 17921361

[21] Lim JS Mietus-Snyder M Valente A Schwarz JM Lustig RH. The role of fructose in the pathogenesis of NAFLD and the metabolic syndrome. Nat Rev Gastroenterol Hepatol. (2010) 7:251–64. 10.1038/nrgastro.2010.41 20368739

[22] Rutledge AC Adeli K. Fructose and the metabolic syndrome: pathophysiology and molecular mechanisms. Nutr Rev 65 (6 Pt 2). (2007) S13–23. 10.1301/nr.2007.jun.S13-S23 17605309

[23] Lanaspa MA Ishimoto T Li N Cicerchi C Orlicky DJ Ruzycki P. Endogenous fructose production and metabolism in the liver contributes to the development of metabolic syndrome. Nat Commun. (2013) 4:2434. 10.1038/ncomms3434 24022321 PMC3833672

[24] Taskinen MR Packard CJ Borén J. Dietary Fructose and the Metabolic Syndrome. Nutrients. (2019) 11:1987. 10.3390/nu11091987 31443567 PMC6770027

[25] Andres-Hernando A Jensen TJ Kuwabara M Orlicky DJ Cicerchi C Li N. Vasopressin mediates fructose-induced metabolic syndrome by activating the V1b receptor. JCI Insight. (2021) 6:e140848. 10.1172/jci.insight.140848 33320834 PMC7821599

[26] Zhang DM Li YC Xu D Ding XQ Kong LD. Protection of curcumin against fructose-induced hyperuricaemia and renal endothelial dysfunction involves NO-mediated JAK-STAT signalling in rats. Food Chem. (2012) 134:2184–93. 10.1016/j.foodchem.2012.04.026 23442673

[27] Wang W Ding XQ Gu TT Song L Li JM Xue QC. Pterostilbene and allopurinol reduce fructose-induced podocyte oxidative stress and inflammation via microRNA-377. Free Radic Biol Med. (2015) 83:214–26. 10.1016/j.freeradbiomed.2015.02.029 25746774

[28] Kang LL Zhang DM Ma CH Zhang JH Jia KK Liu JH. Cinnamaldehyde and allopurinol reduce fructose-induced cardiac inflammation and fibrosis by attenuating CD36-mediated TLR4/6-IRAK4/1 signaling to suppress NLRP3 inflammasome activation. Sci Rep. (2016) 6:27460. 10.1038/srep27460 27270216 PMC4897702

[29] Yang YZ Zhao XJ Xu HJ Wang SC Pan Y Wang SJ. Magnesium isoglycyrrhizinate ameliorates high fructose-induced liver fibrosis in rat by increasing miR-375-3p to suppress JAK2/STAT3 pathway and TGF-beta1/Smad signaling. Acta Pharmacol Sin. (2019) 40:879–94. 10.1038/s41401-018-0194-4 30568253 PMC6786319

[30] Krause N Wegner A. Fructose Metabolism in Cancer. Cells. (2020) 9:2635. 10.3390/cells9122635 33302403 PMC7762580

[31] Luo Y Xu W Chen H Warburton D Dong R Qian B. A novel profibrotic mechanism mediated by TGFβ-stimulated collagen prolyl hydroxylase expression in fibrotic lung mesenchymal cells. J Pathol. (2015) 236:384–94. 10.1002/path.4530 25779936 PMC4457642

[32] Kahata K Dadras MS Moustakas A. TGF-β Family signaling in epithelial differentiation and epithelial-mesenchymal transition. Cold Spring Harb Perspect Biol. (2018) 10:a022194. 10.1101/cshperspect.a022194 28246184 PMC5749157

[33] Ahmed N Furth AJ. Failure of common glycation assays to detect glycation by fructose. Clin Chem. (1992) 38:1301–3. 10.1093/clinchem/38.7.1301 1623595

[34] Schalkwijk CG Stehouwer CD van Hinsbergh VW. Fructose-mediated non-enzymatic glycation: sweet coupling or bad modification. Diabetes Metab Res Rev. (2004) 20:369–82. 10.1002/dmrr.488 15343583

[35] Downs I Liu J Aw TY Adegboyega PA Ajuebor MN. The ROS scavenger, NAC, regulates hepatic Vα14iNKT cells signaling during Fas mAb-dependent fulminant liver failure. PLoS ONE. (2012) 7:e38051. 10.1371/journal.pone.0038051 22701598 PMC3368940

[36] Tenório M Graciliano NG Moura FA Oliveira ACM Goulart MOF. N-Acetylcysteine (NAC): impacts on human health. Antioxidants (Basel). (2021) 10:967. 10.3390/antiox10060967 34208683 PMC8234027

[37] Epstein Shochet G Brook E Bardenstein-Wald B Shitrit D. TGF-β pathway activation by idiopathic pulmonary fibrosis (IPF) fibroblast derived soluble factors is mediated by IL-6 trans-signaling. Respir Res. (2020) 21:56. 10.1186/s12931-020-1319-0 32070329 PMC7029598

[38] Hernandez-Diazcouder A Romero-Nava R Carbo R Sanchez-Lozada LG Sanchez-Munoz F. High fructose intake and adipogenesis. Int J Mol Sci. (2019) 20:2787. 10.3390/ijms20112787 31181590 PMC6600229

[39] Monzavi-Karbassi B Hine RJ Stanley JS Ramani VP Carcel-Trullols J Whitehead TL. Fructose as a carbon source induces an aggressive phenotype in MDA-MB-468 breast tumor cells. Int J Oncol. (2010) 37:615–22. 10.3892/ijo_00000710 20664930 PMC3267577

[40] Nakamura H Takada K. Reactive oxygen species in cancer: current findings and future directions. Cancer Sci. (2021) 112:3945–52. 10.1111/cas.15068 34286881 PMC8486193

[41] Sena LA Chandel NS. Physiological roles of mitochondrial reactive oxygen species. Mol Cell. (2012) 48:158–67. 10.1016/j.molcel.2012.09.025 23102266 PMC3484374

[42] Okon IS Zou MH. Mitochondrial ROS and cancer drug resistance: implications for therapy. Pharmacol Res. (2015) 100:170–4. 10.1016/j.phrs.2015.06.013 26276086 PMC4893310

[43] Gorrini C Harris IS Mak TW. Modulation of oxidative stress as an anticancer strategy. Nat Rev Drug Discov. (2013) 12:931–47. 10.1038/nrd4002 24287781

[44] Weng Y Fan X Bai Y Wang S Huang H Yang H. SLC2A5 promotes lung adenocarcinoma cell growth and metastasis by enhancing fructose utilization. Cell Death Discov. (2018) 4:38. 10.1038/s41420-018-0038-5 29531835 PMC5841403

[45] Wu L Derynck R. Essential role of TGF-beta signaling in glucose-induced cell hypertrophy. Dev Cell. (2009) 17:35–48. 10.1016/j.devcel.2009.05.010 19619490 PMC2722039

[46] Sullivan DE Ferris M Pociask D Brody AR. The latent form of TGFβ1 is induced by TNFα through an ERK specific pathway and is activated by asbestos-derived reactive oxygen species in vitro and in vivo. J Immunotoxicol. (2008) 5:145–9. 10.1080/15476910802085822 18569384

[47] Shi M Zhu J Wang R Chen X Mi L Walz T. Latent TGF-β structure and activation. Nature. (2011) 474:343–9. 10.1038/nature10152 21677751 PMC4717672

[48] Conroy KP Kitto LJ Henderson NC. αv integrins: key regulators of tissue fibrosis. Cell Tissue Res. (2016) 365:511–9. 10.1007/s00441-016-2407-9 27139180 PMC5010580

[49] Song L Chen TY Zhao XJ Xu Q Jiao RQ Li JM. Pterostilbene prevents hepatocyte epithelial-mesenchymal transition in fructose-induced liver fibrosis through suppressing miR-34a/Sirt1/p53 and TGF-beta1/Smads signalling. Br J Pharmacol. (2019) 176:1619–34. 10.1111/bph.14573 30632134 PMC6514378

[50] Kasai H Allen JT Mason RM Kamimura T Zhang Z. TGF-beta1 induces human alveolar epithelial to mesenchymal cell transition (EMT). Respir Res. (2005) 6:56. 10.1186/1465-9921-6-56 15946381 PMC1177991

[51] Batlle E Sancho E Francí C Domínguez D Monfar M Baulida J. The transcription factor snail is a repressor of E-cadherin gene expression in epithelial tumour cells. Nat Cell Biol. (2000) 2:84–9. 10.1038/35000034 10655587

[52] Boutet A De Frutos CA Maxwell PH Mayol MJ Romero J Nieto MA. Snail activation disrupts tissue homeostasis and induces fibrosis in the adult kidney. EMBO J. (2006) 25:5603–13. 10.1038/sj.emboj.7601421 17093497 PMC1679761

