# Supplementary material for: Fructose Induces Pulmonary Fibrotic Phenotype Through Promoting Epithelial-Mesenchymal Transition Mediated by ROS-Activated Latent TGF-β1
Source: Front Nutr. 2022 May 27;9:850689. doi: 10.3389/fnut.2022.850689 (PMC9197188; doi:10.3389/fnut.2022.850689)
Supplement: Supplementary file 1 [file Presentation_1.pdf]

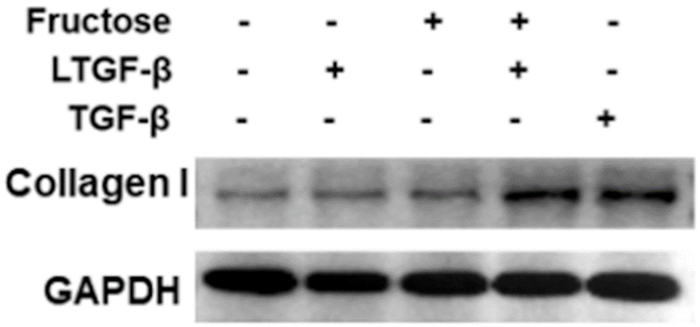

Figure S1 Effect of fructose on fibrosis-related proteins in BESA-2B cells induced by latent TGF-β. Western blot analyzed the expression of collagen I in the lysates of BESA-2B cells treated under the indicated condition. Representative gels were shown, and the experiment was repeated at least three times.

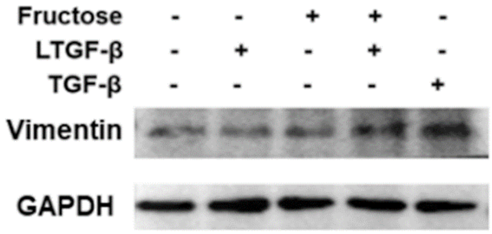

Figure S2 Effect of fructose on the latent TGF-β induced EMT process in BESA-2B cells. Expression of Vimentin in the lysates of BESA-2B cells treated under the indicated condition was assessed by Western blot. Representative gels were shown, and the experiment was repeated at least three times.

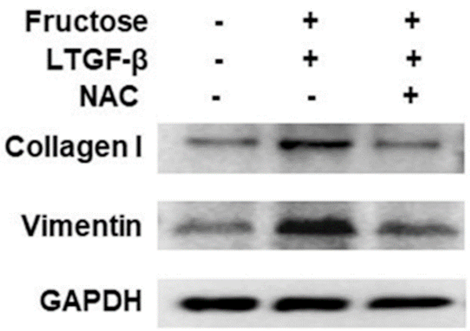

Figure S3 Effect of NAC scavenging fructose-induced ROS on the fibrosis and EMT process of BESA-2B cells treated by latent TGF-β. BESA-2B cells were treated under the indicated treatment for 24 hours, and then the expression of fibrosis-related protein (Collagen I) and EMT-related protein (Vimentin) was assessed by Western blot. The experiment was repeated at least three times.

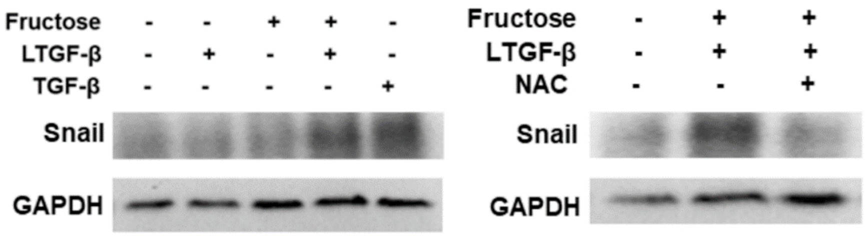

Figure S4 Effect of fructose on the TGF-β/ Snail1 expression in latent TGF-β-induced BESA-2B cells. BESA-2B cells were treated under the indicated condition for 24 hours, and then western blot analyzed the snail protein levels. The experiment was repeated at least three times.

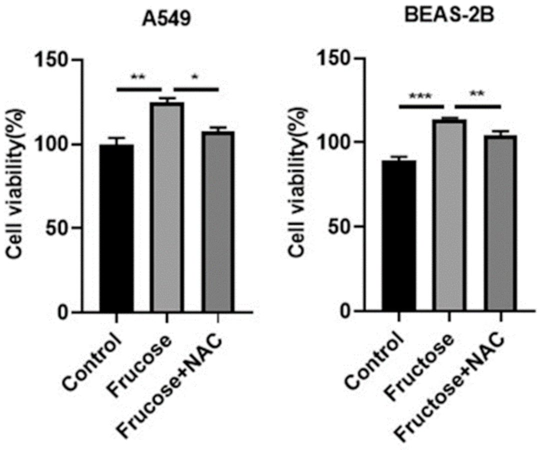

Figure S5 NAC can inhibit the effect of fructose on the proliferation of human lung epithelia cells by scavenging ROS. A549 and BEAS-2B cells were cultured by fructose (10 mM) with or without NAC (5mM) for 24 hours, and then a CCK-8 assay was performed to detect the cell viability. Data were expressed as Mean ±S.E.M (n = 3) and analyzed using one-way ANOVA followed by Tukey's test. *p < 0.05, and **p<0.01 vs 0 mM fructose.
